# Care Networking: A Study of Technical Mediations in a Home Telecare Service

**DOI:** 10.3390/ijerph10073072

**Published:** 2013-07-22

**Authors:** Gonzalo Correa, Miquel Domènech

**Affiliations:** Departament de Psicologia Social, Universitat Autònoma de Barcelona, Barcelona 8014, Spain; E-Mail: miquel.domenech@uab.cat

**Keywords:** older people, technical mediation, interdependence

## Abstract

This article examines the processes of technical mediation within familial care networks based on a study of home telecare targeted at older people. Supported by contributions from the actor—network theory as part of the social psychology of science and technology, these processes of technical mediation are analyzed using a qualitative approach. The data were gathered through six focus groups and four in-depth interviews; the participants in the study included users, relatives and formal carers. Thematic analysis techniques encompassing the information were used, revealing the effects on the patterns of caring relationships. The results show the interplay between presence-absence made possible by the devices; the two-way direction of care between the older people and the artifacts; and the process of sustaining care using the technology. We conclude that care should be seen as a socio-technical network where technology plays an active role in sustaining family relationships.

## 1. Introduction

Family plays a central role in the care of older people. Over the course of the past few decades, especially in Europe, family lifestyles have undergone major transformations that hinder caregiving practices, including a drop in family size, an increase in geographic family mobility and a massive incorporation of women into the job market. These changes have been suggested as leading to a decrease in the capacity to provide care, something that has coincided with an increasing number of older people. Although this does not necessarily have to involve an increase in the need for care support, many Western governments have foreseen a risk for social welfare, even speaking of a future crisis of care [1].

However, the modern family still plays a prominent role in caring for those members that need help or attention [2,3,4]: relatives are still the main actors for both hiring services (traditional and/or innovative), and providing care and economic resources [5]. Family members are, in short, the main caregivers in informal care (a provision of care without any economic compensation for the aid offered). The scope and diversity of this kind of care has been discussed in numerous studies [6,7]. Informal care is regarded as an important predictor of institutionalization given that good family support (in the broad sense) can avoid or delay this process [8,9,10]. The successes and failures of care practices depend on the qualities of family networks, which may or may not be limited to relatives. Several studies report the difficulties its members may suffer in situations where one of them endures chronic illness or severe dementia [11,12,13,14,15]; the most frequently mentioned negative effects are the sense of loneliness, isolation, boredom and frustration [14,16,17]. Even if these studies are not representative of all care practices, they still highlight the vulnerability of informal networks exacerbated by changes in the constitution of families.

Despite Western governments’ attempts to promote formal care, there is a series of obstacles linked to economic issues (high costs), demographic considerations (a low number of young workers willing to take on these jobs) and issues regarding respective political administrations (state reforms unable to keep up with the accelerated pace of the transformation of the family, and the increasing incorporation of women into the workforce) [4]. As a consequence, it can be said that there is a lack of support for caregivers overcome by the demands of the role [18], which means that the people who truly need care are not receiving it. In this sense, it is acknowledged that social services and family exchanges play a fundamental role in the welfare of those involved in care [2].

As a complex practice, care involves an assortment of actors, processes, resources and regulations, as well as diverse, disparate forms of knowledge [2,19,20]. This has led to questioning the simplistic ways of addressing caring relationships, such as the ones implicit in the triangular model of care (users-relatives-service providers) [21], and to instead focus on another more polygonal model, which includes not only the traditional actors, but also professional caregivers, institutions, the market and others [22]. This would confer a more marked interrelational nature on care [18].

Technology has come onto the scene as a possible solution to the apparent care crisis. Several studies have paid special attention to the role it plays in the care of older people, stressing its active role in caregiving processes [17,23,24,25,26,27,28,29,30,31,32]. Different devices have appeared—and continue to appear—that have become new aids in fulfilling this job [33,34]. Home telecare appears as a principal solution to this demand. The version of telecare we refer to is the Spanish one: a monitoring system that involves the installation of a domestic terminal through which the user can communicate with an alarm center 24 h a day. All users have to do is to press the red button on their terminal, or one on a pendant around their neck, to get in touch with a teleoperator. They can then ask for immediate help or talk about anything, even if it’s not particularly urgent. If help is needed, the teleoperator will call the most appropriate resources (relatives, ambulances, firefighters, *etc*.). Teleoperators have access to a database that contains all the medical and personal details of the user to decide which resources are the most suitable.

Among a variety of benefits, home telecare service is considered a good solution for the care of older people due to both cost reduction [35] and a positive view of active ageing [23,24,36,37]. Occasionally, telecare is presented as a decisive step towards replacing residential forms of care [38,39,40] and the promotion of “ageing in place” policies [41].

To address how technologies relate to people in care practice, we propose to use the concept of technological mediation. Latour [42] defines mediation as something that happens that is neither fully the cause nor fully the consequence; something that occurs without being either totally a means or totally an end. The distinction between subject and object becomes moot, since these entities are dissolved into networks of mediations in which different agents take part without necessarily having to be human beings [43]. These agents are mediators given their capacity to translate and let themselves be translated by others. Translation is viewed as a displacement whose outcome is the creation of a new bond which previously did not exist, and which prompts an ontological change in the agents in the specific network where the shift takes place, thus creating new entities. Indeed, translation can be defined as all the negotiations and acts of persuasion through which the actors manage to attain the adhesion of other actors, reorganizing the entities and their relations, and thus shaping a network [44].

In view of the dissolution of the subject and object, and in view of the redistribution of agency (which used to be a solely human monopoly), the concept of *actant* emerges, extracted from Greimas’ semiotics [45]. Greimas refers to those entities (previously subjects and objects) that try to translate each other, remarking that someone or something’s capacity to operate always refers to a relational network [27]. Thus, the role of *actant* cannot be attributed to a subject or object a priori, since the action is always the result of the ties that emerge among the different components of the network. As Latour notes, action is a property of associated entities [42], and objects thus take on an active role. To Latour, a mediating entity is one that is capable of producing translations in the other actors in the network.

In this paper, we analyze the role of technology in family care relationships. Based on a qualitative study of home telecare service, we argue the mediating quality of technology; that is, the effects it produces in family interactions. Far from applying an aprioristic view in terms of good or bad—which used to be the consequence of a deterministic analysis—we state that the technology is not neutral [46] when the evidence is considered. To demonstrate this, we analyze various forms of technical mediation appearing in the interactions of carers, home telecare users and relatives.

## 2. Experimental Section

### 2.1. The Aim of the Study

The literature surveyed shows the predominant role of the family in the care of older people despite the increasing inclusion of technology and other agents in caregiving practices. The purpose of this study is to explore the processes of technical mediation in the family setting in relation to home telecare targeted at older people. More specifically, the study aims to boost our understanding of the day-to-day relationships between technological devices, older people and their families.

### 2.2. Method and Design

This study was part of a research project focused on the inclusion of home telecare in the caregiving processes targeted at older people in Spain. This project received financing from the Ministry of Science and Innovation, and was entitled “Science, technology and the care of dependence: the transformation of care in the knowledge society.” Although the project comprises data from participant observation, semi-structured interviews and focus groups, we have focused the analysis on a group of data from the first period of the research (2009) to achieve the objective of this study, which corresponds to 57 people as informants.

Participation was voluntary, and we gathered the respective informed consent forms in which the subjects were told about the anonymity of the participants, the purpose of the study and the reserved use of the information, and which gave their authorization to record and make transcriptions of the events. The different sessions were held in both Spanish and Catalan, and the use of these languages was respected for both the transcription and the analysis of the material.

### 2.3. Selection of Participants

All 57 participants were asked to participate because of their status as either users of home telecare, family members who cared for home telecare users or formal carers. For older people, the requirement was that they lived in their own homes and had been using the service for at least one year; for the family caregivers, they had to be in charge of older people; for formal carers, they simply had to work in a home telecare service regardless of the job they performed. After the four interviews and the six focus groups it was concluded that we had reached a point of saturation, and that there was rich enough data to explore the issues at hand in this study.

### 2.4. Data Collection

We employed a purposive sample [47], which ensures the necessary diversity of the participants; this provides a structured sample instead of a random one. In this sense, we developed a sampling strategy based on the topic at hand, which basically aimed to ensure that the participants had different geographical origins, ages, genders and positions in the relation of care.

First, four in-depth interviews were held with older people who use the home telecare service and their respective relatives [48,49,50] to explore the topics of care relationships and technological frames in the family. In the interviews, we further explored the effects of telecare in the care network by inquiring into the following topics: (i) how caring relationships are defined and described; (ii) how they shape and establish relations of dependence, independence and interdependence, as well as processes of autonomy and/or heteronomy; (iii) forms of socialization that take place around these networks; (iv) the use of time and space; (v) the most significant relationships established with artifacts; (vi) the effect on the interpersonal relationship of older people and carers; (vii) the changes that have taken place in caring relationships as a result of the home telecare device; and (viii) the role assigned to technology in caring relationships.

Later, six focus groups were used, which included older people and informal and formal carers, with a total of 53 participants. The focus groups were held in six towns in Catalonia—Badalona, Barcelona, Granollers, Igualada, Mataró and Vic—in 2009, and they yielded approximately 12 h of recorded material (Table 1).

**Table 1:** Composition of the focus group.

Town	Date	Participants	Composition
Igualada	27/04/2009	8	Older people
Mataró	11/05/2009	8	Formal carers
Badalona	14/05/2009	11	Older people/Informal carers/Formal carers
Granollers	23/09/2009	9	Older people
Vic	01/12/2009	8	Formal carers/Informal carers
Barcelona	15/05/2009	9	Formal carers

The participants were asked to talk about their experiences and opinions regarding telecare. The main goal was to ascertain the social perception of how telecare services promoted autonomy, with the care dimension as the backbone. Unlike other researchers who have used this technique to measure attitudes towards technology or services [25,26], the focus groups were envisioned more as informal talks guided by a specific task [51]. This enabled the participants to share their different points of view, mining their immediate experiences while also producing discussion and debate.

### 2.5. Analysis

For this analysis we used literal transcriptions of the focus groups and in-depth interviews, which were verified with the corresponding recordings and later analyzed using thematic analysis techniques [44]. The job of analyzing entailed an initial pre-analysis stage [52] in which the entire corpus was studied in detail, which enabled us to identify a series of subjects referring to caring relationships. Later, excerpts from the material were chosen in which the actors reported on different topics related to technology and care’s relationship; these were taken as units of meaning following the procedure used by Fex and colleagues [53]. In these excerpts, meanings were identified that revealed the different directions and trajectories taken in the family care network. Based on this identification, we began to establish a series of thematic fields in direct relation with the themes that had initially been identified, each of which was named with their most salient characteristic in mind.

The successive interpretations enabled us to modify these themes, and with them the organizing criteria and location of the different units of meaning. Following Coffey and Atkinson [54], we identified a series of key themes and patterns by assigning labels to the data which kept in mind the words of the participants. Setting up these labels or codes helped us to group them into categories based on their similarities and differences: the first step involving decontextualization was necessary to make way for a second step involving recontextualization [54]; this, in turn, led to the merger of different units of meaning that arose in the interviews and focus groups, bearing in mind the objectives of the research and the novel aspects emerging from the discourses analyzed. The categories with similar content were grouped together and summarized. They were then adjusted and reformulated in a lower number of categories through an inductive, repetitive process, which implied an exchange between the data, and consultations of the literature on the subject and the conceptual framework proposed to address technical mediation [19]. Finally, the emergent themes were described in depth based on a semiotic understanding of human (and non-human) relations, which views them as “webs of meanings” [55]. In this sense, the analysis consisted of unraveling these meanings and their scopes, further deepening the existing relations between the statements and the emerging categories, and debating the paradoxical aspects that appeared in the discourses of the participants. This enabled us to enrich the analysis of the data to reach a series of findings.

## 3. Results and Discussion

### 3.1. Findings

Below we present the study’s findings based on identification of the ties existing among older people, their relatives, formals carers and telecare artifacts. The following dimensions were examined: the presence-absence made possible by the devices; the two-way direction of care between older people and artifacts; and the interrelational and interdependent nature of the network of care. We present the home telecare devices as one more actor in the care network; for that, we need to describe how the devices play a part in a technical network of family care.

#### 3.1.1. The Presence of Others through the Presence of Artifacts

In care mediated by technology, a new agent joins the family: a set of artifacts whose aim is to guarantee the presence of others. The active role of the technological agents (pendant alarm and terminal) in the home telecare service can be seen in the processes of mediation, which make it possible for the family members to remain present even in their absence. Although relatives are not at home, they can rest assured knowing that if something happens they will be notified.

*P1: Well, peace of mind, sure! Of course! If there is no one at home and we see that grandma is wearing the pendant alarm in case she falls, or for any reason [...], I don’t know. Or, I mean, or that, even if she gets dizzy, something that she herself might find, just push the button and that’s it. I mean not [...]. And even for her, too. For her […] at least every month and a half or two months, they call from the device to see if everything is working properly, I mean even she feels less alone in case anything happens, I mean [...]. I see that it gives us a lot of peace of mind.*

(Relative in focus group 6, lines 224–229).

The silent presence of the artifacts brings to the home the possibility for the constant presence of others. It is this which leads to the construction of security in terms of peace of mind and trust as a stabilized result of a series of arrangements [56]. In this way, the artifacts are not mere intermediaries that connect the user to an alarm center; their purpose is not limited to this (although this is the prescribed script), but their presence provides the possibility of immediate connection for the human care agent. The absence of the family member translates into the presence of the pendant alarm and home terminal. Bruno Latour termed this process as “delegation”. By virtue of it, an object replaces an actor and redistributes the relative ordering of presence and absence: actors can be remote in time and space yet simultaneously active and present [42]. This is certainly a virtual presence. According to Lèvy, when a person, a community or an act are virtualized, they are placed “out there”, they are deterritorialized. They suffer a kind of detachment from the ordinary physical or geographical space and from the temporality linked to the clock and the calendar. It cannot be said that they are completely independent of the space-time reference, as they always have to materialize here or elsewhere, now or later, but they subvert the ordinary meaning of presence and absence [57].

Even though these artifacts appear as simple intermediaries which connect the user to the alarm center, their place in the network of care enables new movements to be established. The mediation of the process of keeping someone company is an important aspect of home telecare in constructing a sense of security. The device guarantees that both the family members and users will not be alone; their presence boosts the security of all parties, distributes responsibility and expands the possibilities for care as soon as it sets up the connection with other agents. The inclusion of the pendant alarm and home terminal into the family setting (in the generic sense) and the home (in the specific sense) enables potential risks to be prevented by guaranteeing the presence of others (family members, service providers, health-care services, *etc*.) in their absence. This process is sustained precisely by the interplay between absence and presence, in which the circumstantial absence of family members is translated by the artifacts into a permanent presence.

*P4: You see, it’s for the person when they’re alone and as long as that person is there, it is also a resource that you need: telecare and that’s all. The children are at ease and the caregivers are as well, because you know [...].*

(Woman user in focus group 4, lines 1,638–1,640).

As the above quote reveals, the presence of the artifact must be constant when the person is alone and when the caregivers are with them in order to sustain the care in the way it is supposed to. The issue is not only whether or not it is used in emergencies—its usefulness lies in its mute, permanent presence. This silent participation of the artifact enables the home to be connected to a series of resources, and for users to feel accompanied even when alone, thus making them feel more secure. As a result of this first translation, the artifacts make the constant presence of the family possible.

Communication with the family is often mediated through objects. The users communicate with the alarm center before they communicate with their family members, shifting the role of the family in resolving emergencies or urgent situations.

*P2: Sometimes they say: “Mama, but if something happens to you call us first, eh? Don’t be silly!” And I say: “Good lord, I have to call you first?” I’ll call the Red Cross and be done with it. “Oh, but it’s a snap to call, Mama.” Fine, but look.*

*I: So you would call the Red Cross before calling your children?*

*P2: Of course I would because you can’t imagine the fuss it would raise if I call them, but if I call the Red Cross you see they’d come and do what they have to do, and then I’d let them know that I’m there, right?*

(Interaction between the interviewer and a woman user in focus group 2, lines 919–927).

The silent presence of objects enables several different actors to exchange, interact and mobilize, shifting their initial positions and shaping a new kind of network. As expressed in the excerpt above, home telecare envisages a shift in how emergencies are handled within the family, displacing the family members’ prescribed function. The tension that arises between the daughter’s request and the mother’s alignment with the purposes of the device reveal a second translation: the shift from being a mother to being a user.

This translation implies a change in the quality of the agents, and with it a reconfiguration of the entire network, each with displacements. The commotion that might occur if she calls her daughter translates into the technical know-how of the home telecare operators. This translation is necessary for other agents to enter the home, as well as for the presence of the family to be constant despite their absence. At first glance, the above quotes certainly seem to indicate that the system works very well in getting the family out of the way of the older people, as well as in making the family absent. Our point here is that this is only a formal absence, and that the system makes it possible because the family’s presence becomes delegated into the artifact.

#### 3.1.2. Another One to be Cared for

All caregiving practices entail a two-way relationship in which the person being cared for cares for their caregivers; in the case of the home telecare, the artifacts play a very important role in this interchange of care, enabling the caregivers to be cared for. In our corpus we have identified how users take care of their caregivers, artifacts included. As we shall see, caring for the latter is an important task for the service to achieve.

A clear expression of how users care for others is through trying to resolve the situations themselves without bothering them. This implies evaluating the damages, and considering whether or not to push the alarm button based on this evaluation.

*U1: [O]ne day, not too long ago, a month or month and a half ago, I fell here. I fell, I tried to support myself on this chair and I got up and of course [...] I put my weight on the chair, but when I did it the chair fell, of course, so I couldn’t, with my weight, the chair fell down over that way and I fell the other way. Whew, my arm still hurts. I said, “God, God, how am I going to get up now...? Shhh, stay calm, stay still, stay here a minute, calm down, calm down, relax,” I said, [...]. And after resting a while I was sitting down, resting on this wall here, I said, “Ok, everything hurts but no, I haven’t broken anything because the fall was soft. No, so get up,” and of course I wasn’t going to call because you’re not going to stir things up just for something like this. So I got up, I sat down for a while the way I am now, I calmed down, let’s see what’s going to happen to you tonight [...]. Because, of course, after the fall you’re all worked up [...].*

(Interview 1, lines 134–148).

In home telecare service, the possibility of “not disturbing” others and evaluating when one should do so is possible thanks to the presence of the artifacts. The care of others takes place through the particular use the person gives to the artifacts. In the solitude of the home, people and pendant alarms encounter each other; not pushing the button translates into not bothering other absent people. Since the family members and telephone operators are far away, not pushing the button on the pendant alarm is one of the forms of telecare that the person being cared for uses to care for their carers.

But, furthermore, the presence of home telecare devices brings new agents to be cared for. The home terminal and the pendant are not only good caregivers; they are also the object of care by users and their relatives. Caring for the object implies directly caring for others and for oneself. The users are accustomed to receiving periodic phone calls (every 15 days or monthly) which fulfill a twofold purpose: to check on their state of health; and to determine whether the home terminal is working properly.

*P1: Yes, yes, yes. And she says: “I got a call from the Red Cross.” Or then she tells me, about a month later, she says: “I got a call to check on it. I got a call from the alarm center to check to see whether…” She says it. [...] Well, you know, if the device is working. They tell her what to press and then that’s all. So she tells us about it if we are not at home.*

(Relative in focus group 6, lines 345–351).

This movement is part of the transition from an older person to a user. Being a user means accepting the accomplishment of a set of actions, including the care for the artifacts. The protocol stipulates periodic calls and this repetition maintains the position, highlighting the active role of the people being cared for in this maintenance. The same happens with those agents that participate in the care of older people, now mobilized to care for the devices. As a result, older people have their pendant and home terminal, and the latter have its user. The active nature of the users provokes a particular responsibility through which care is sustained into the care process. For care mediation to be effective, it is necessary to take care of the artifacts. In the system, users play a key role in monitoring the performance of the pendant alarm and home terminal. Malfunction of the devices can be bothersome to the users in a minor sense, and in the major sense it can be a risk that can leave them totally cut off. Certain flaws that trigger discomfort are recurrent.

*P4: Sometimes it used to make noise.*

*I: The device? It made noise?*

*P4: Yes, well I came here and they fixed it for me, well they told me, they said: “If it makes noise push the green button,” but I didn’t remember that they’d told me that.*

*I: Ah!*

*P4: And, I don’t know, later it might happen again, because that noise is awful, eh?*

(Interaction between researcher and user in focus group 2, lines 1,105–1,114).

The encounter between the agents in certain conditions of uncertainty leads to varied actions: there are attempts to respond to the unknown, promote particular relations with the artifacts and step up the mobilization of other agents. When the home terminal breaks the silence, when it ceases to be what it used to be and is no longer recognized by its behavior, the actions of all the actors involved revolutionize, and the *a priori* meaning of their behaviors destabilize. These situations reveal our current lack of knowledge regarding the mediating role of the device given its stabilization and presentation as a mere intermediary. An effort is needed by the family members and users to maintain the stability of the artifacts. Just like humans, the composition and behavior of objects are variable. Users’ care of the pendant alarm and home terminal reveal the two-way direction of the caregiving practice, in which the person being cared for must also take care of the thing that helps them to be cared for—in this case an artifact. These caregiving practices on devices, expressed in the periodical phone calls and alerts in case of flaws, entail permanent action by the human caregiving agents, whose purpose is nothing more than guaranteeing certain conditions of stability so that the pendant alarm and home terminal can work as mediators.

#### 3.1.3. Technology as a Support for Care

The presence of artifacts in the household plays an active role in supporting care. More than the actual caring per se, their role is to support the caring relations of other agents. In this sense, their inclusion exemplifies the socio-economic transformation of the family in recent decades: as an actor that contributes to a new configuration of older people’s care; one which allows more actors to be included and promotes changes in the actions of present agents.

*P4: I don’t think that it replaces others, which you said that it brings compared to other forms of care. I don’t see it as a, I mean, this telecare changes ways of caring for people, because it replaces, I think that telecare is a consequence of the fact that the way care is provided has changed. Now we women work outside the home […] and we’re not there. Before [...] the entire family lived together, we were all together. Now we’re not; everyone has their own house, their schedule, their own obligations and we all have to look for our own solutions to deal with everything; to be able to fill the gaps; no, to be able to get through daily life better. But I don’t [...] I don’t think that they offer anything that the work, I mean family, setting could offer in a much better way.*

(Relative in focus group 4, lines 439–447).

In view of the dispersion of the family and the obstacles and solutions formulated (“we have to look for our own solutions”), home telecare service appears as a technical network that helps maintain current agents’ caregiving conditions. It is true that we have shown several changes in familial situations, but it is equally true that telecare becomes a way to make it possible for some features of pre-existing relations to continue. In this vein, the device takes on a central role in the family architecture (considering the transformation of family care in today’s context) by promoting the constitution of a socio-technical network of care.

*P6: In fact, they have a social commitment as children. “I’m calling you not because I don’t want to do my job but because you’re also part of the chain.”*

(Formal carer in focus group 5, lines 304–305).

The responsibility and commitment of the older people’s children is key to this process. Being “part of the chain” means accepting the new shape, and ultimately the reconfiguration, of the family and technology in the care of older people. At the same time, it reveals the impossibility of care without the presence of the family, and in doing so it demonstrates the device’s function of support within the network of care. Even though home telecare appears as a consequence, or response to, the new family configuration, more than anything it is a producer of new forms of interaction and treatment for family caregiving. However, for this to be possible, the goals of all the actors involved must be translated. This leads to the establishment of negotiation relations, which directly involve older people and their relatives.

*P6: You find, percentage-wise, that the majority of users have this problem when a family member requests the service. If the user requests the service, you know that they’re going to use it. However, when a son or daughter requests it, which happens a lot, either by telephone or they come to the office to ask for information, you end up saying look, the service [...] I want it for my father or my mother, but do they want it? No, no. So, let’s see. The service is for them, right? The person who has to want it is them, because if you want it we’re going to set it up and they’re going to say, “Yes, yes, yes,” so we shut up, so that we leave them alone, and when we leave they’re going to take it off or leave it on top of the nightstand. And this percentage is on the rise [...]. If the user him- or herself requests the service, it’s to be expected more than [...].*

*P1: Than when it seems that the children want to tell them what to do?*

*I1: Right, of course. And how do you deal with this?*

*P2: The only thing that happens is that older people probably don’t even want to bother their children, right? So the children, and here things change, set up this device to keep an eye on them. They think their parents are better watched over. But there are people who don’t want it and others who do. The people who don’t want it, when something happens, I have a user who this happened to and then she did start wearing it. But if nothing happens to them, I mean if nothing happens to them, well [...] I mainly think that it is the children who want to keep an eye on their parents and that’s why they get it.*

(Interaction between researcher and formal carers in focus group 3, lines 331–352).

In the midst of these negotiations, the artifacts silently operate as connectors between the several interests. The discussion around the introduction of home telecare translates the actors involved, allowing their goals to be brought to the forefront while producing a new collective at the same time. Pendant alarms and home terminals are mediators because they have the ability to align the different interests at stake: relatives are worried about the welfare of older people living alone; older people want to be quiet and maintain their autonomy; home telecare services want to obtain more customers, and so on. The negotiation is a process where the beginning and end are not determined beforehand. It implies several changes: the actors modify their initial positions; conflicts emerge; acts of persuasion, confrontation or acceptance appear in the network. But the result is unpredictable; the telecare artifacts can or cannot remain at home. Involvement implies all the parties’ acceptance of wanting to be part of the new set-up being proposed. This initial process is necessary for the telecare network be formed. When older people accept its introduction, the previous network of care is translated to a new composition thanks to the pendant alarm and the home terminal.

### 3.2. Discussion

The verbatim seem to point to a view of technology as something not neutral. We mean that technology produces effects and helps to shift and (re)build networks; that is, family life changes once technology is introduced. This is precisely what we have tried to show with the presented data. In order to understand these effects, we have proposed the notion of mediation.

Our point of departure was to conceive caregiving practices as complex relations involving interrelation, interaction and interdependence among human actors (caregivers, older people, service providers, *etc*.) and a diversity of objects that are an inherent part of the caregiving network (from the materiality of the household to communication technologies, and the specificity of the pendant alarm and the home terminal in the home telecare service). In this way, we can define care as a network of mediations, one of whose prominent agents is the set of technical elements which are inherent in it; hence the importance of studying the processes of technical mediation in familial caring relationships and our proposal to do so, given that it is the result of the combined interests and goals of the different actors. In this sense, care is mediated by technologies, producing a socio-technical network in which a diversity of actors takes part—both human and non-human. The relations established among the participants are characterized by interdependence as part of an ecology of action that stabilizes and destabilizes relations, abilities and inabilities [27], and that sustains and reassembles the care in a constantly dynamic interplay.

Following Latour [42], there are different forms of mediation; that is, there are several ways to produce alliances between human and material actors. In our research, we identify three mediations acting in and defining the care relations: the presence-absence of actors (delegation); the two-way direction of care (translation of goals); and the interrelational and interdependent nature of the network of care (composition).

One of the most significant effects of technology’s inclusion in care is the interference in traditional notions of it. Care is no longer defined as a two-way practice upheld on the caregiver-cared dichotomy; rather it has become an ecology of multidirectional action in which a range of human and non-human agents take part [27]. As Molyneaux, Butchard, Simpson and Murray [58] state, it is necessary to question the concept of “caregiver,” since its use may have a negative effect on the person being “cared for,” particularly when dealing with a joint effort among the participating agents. In order to avoid the use of this binomial, they propose speaking simply about “caring relationships.”

In this sense, the active efforts of older people, their relatives and the home telecare personnel show how caring relationships are sustained by technology. Care mediated by technology turns into a socio-technical network that folds into a single movement towards protection and care [27,28,29,30,59]. In order for it to work, the agents must act out certain established roles. We can no longer talk about caregivers and the cared for; rather, functional subjects of the home telecare device emerge. The transformation of older people into users is one of the main translations in the socio-technical network. This translation implies letting oneself get involved via the device and thus exchanging competences and goals. Older people become “users” as soon as their actions reflect the guidelines of the device. This translation modifies the distances and relations within the family, displacing responsibilities and distributing new scripts: being a user implies communicating with the central office periodically; ensuring the soundness of the artifacts; wearing the pendant alarm; and, in case of emergency, having the service as the reference instead of family members. Likewise, being a family member implies being “also part of the chain”, and becoming a contact in the circuit of the device’s information. In order for this to occur, both the users and the family members go through the telephone exchange as an *obligatory passage point* [42]. Although it is not the focus of this paper, another line of analysis derived from the translations described refers to autonomy, a specific supposed benefit of home telecare service. While numerous studies have conceptualized autonomy from an individual perspective, and from this approach considered ageing as a gradual loss of this autonomy [36,59,60,61], studies from critical gerontology have defined it as a result of relations of interdependence [12,26,60,62,63]. Our analysis of mediation provides evidence along the same lines. The relation of both autonomy and heteronomy emerging from the care network does not depend on a merely individual issue, nor solutions strictly associated with the context. The important thing to bear in mind is which mediations take place, and therefore what new relations and possibilities emerge, as well as to clearly understand what is being sustained. The quantity and quality of agents needed to promote relations of interdependence tending towards autonomy must be analyzed in light of each unique familial situation and their hybrid relations with the other agents involved.

The inclusion of technical caregiving artifacts in this interdependence grants a central role to the notion of practice and the processes of technical mediation [27,53]. Family configurations are a complex web of actors and actions, movements and distances, which must be taken into account when offering solutions. We can no longer talk about a network of family care and circumscribe it exclusively to the family: it is hybrid and takes shape within a network of networks that possess a major connective potential. Just as the family has readily accepted home telecare as a network (there is a significant process of naturalization, and ultimately of acceptance and inclusion), it also has the potential to incorporate other networks that may transform its goals in relation to providing care. It should come as no surprise that forthcoming generations of older people and their relatives will increasingly and even more readily incorporate more technology into the job of providing care. The presence of these networks, as well as the greater possibilities of producing new additions, mean that more and more agents are joining the game, including family members, neighbors, doctors, firemen, computers, mobile telephones, formal carers, keys, telephone operators, and others. The more actors that join, the more the responsibilities and actions will be distributed.

Care as a socio-technical network has altered space and time, as well as the roles assigned to family members: in the former, distances and response capacities have been turned into a quick and accessible point through a codification system based on the transit of information, leading to environments that are more reliable for action [30]; the latter shows an change in the role of family members, who acquire functional roles as circuits of information at the service of home telecare. Both transformations are made possible by the presence of the artifacts, which are not limited to the pendant alarm and home terminal, but also include telephone exchanges, computers, helmets and thousands of kilometers of cables. The presence and strategic distribution of the artifacts shape a system of protection and vigilance—such control being possible by the cooperation of human and non-human actors.

## 4. Conclusions

The core of our argument rests in the mediating role of technology. Alongside this paper we have shown different realizations of the latter through an analysis of familial changes and continuities enabled by a home telecare service.

Our analysis explains care as an active practice that requires the involvement of many different actors, both human and non-human. Our interest has been to show that these non-human actors are not mere instruments of human agents. On the contrary, this case study has allowed us to appreciate the active role of artifacts in a network of care. Sometimes they act instead of human carers, sometimes they translate previous goals, and sometimes they sustain the very network of care. In all cases, anyway, they have an active role whose outcome is a kind of durability through change.

Finally, we have also questioned traditional notions of care and its role in family settings. As the analysis has evidenced, the mediating activity of artifacts produces several translations in family relationships. In this respect, we should ask what these translations mean in the family setting, and where the family is heading in its alliance with technology. These developments show that the family is changing, and that the technified family is emerging as the possible future norm for family relations. In order to go deeper into the effects of technologies of care, more research is needed to evaluate the extent to which these translations contribute to the welfare of people involved, and to promote the consensual construction of new compositions.

## References

[1] McGlone F., Cronin N. (1994). A Crisis in Care? The Future of Family and State Care for Older People in the European Union.

[2] Bazo M.T., Ancizu I. (2004). El papel de la familia y los servicios en el mantenimiento de la autonomía de las personas mayores: Una perspectiva internacional compararada. Revista española de investigaciones Sociológicas.

[3] Molina Sena C., Meléndez Moral J.C., Navarro Pardo E. (2008). Bienestar y calidad de vida en ancianos institucionalizados y no institucionalizados. Anales de psicología.

[4] Outshoorn J. (2008). The provision of home care as a policy problem. J. Comp. Policy Anal..

[5] Albertini M. (2009). What childless older people give: Is the generational link broken?. Ageing Soc..

[6] Jamieson A., Jamieson A. (1993). Atención informal en Europa. Comparación de Políticas Europeas de Atención a Las Personas Ancianas.

[7] Bazo T., Domínguez-Alcón C. (1995). Los cuidados familiares de salud a las personas ancianas y las políticas familiares. Revista española de investigaciones sociológica.

[8] Durán M., De Miguel J. (1998). La mediación invisible: De las utopías sociales a los programas políticos en materia de salud. El Futuro de la Salud.

[9] Álvarez Hernández J., Sicilia Molina M. (2007). Deterioro cognitivo y autonomía personal básica en personas mayores. Anales de psicología.

[10] Roca Roger M., Úbeda Bonet I., Fuentelsaz Gallego C., López Pisa R., Pont Ribas A.,  García Viñets L., Pedreny Oriol R. (2000). Impacto del hecho de cuidar en la salud de los cuidadores familiares. Atención primaria.

[11] Kuuppelomäki M., Sasaki A., Yamada K., Asawaka N., Shimanouchi S. (2004). Coping strategies of family carers for older relatives in Finland. J. Clin. Nurs..

[12] Montorio Cerrato I., Fernández de Trocóniz M.I., López López A., Sánchez Colodrón M. (1998). La entrevista de carga del cuidador. Utilidad y validez del concepto de carga. Anales de psicología.

[13] Coen R. (2002). Individual quality of life factors distinguishing low-burden and high-burden caregivers of dementia patients. Dement. Geriatr. Cogn. Disord..

[14] Rawlings J., Spencer M. (2002). Daughters and wives as informal care givers of the chronically ill elderly in trinidad. J. Comp. Fam. Stud..

[15] Robinson K., Steele D. (1995). The relationship between health and social support in caregiving wives as perceived by significant others. J. Adv. Nurs..

[16] Chambers M., Ryan A.A., Connor S.L. (2001). Exploring the emotional support needs and coping strategies of family carers. J. Psychiatr. Ment. Health Nurs..

[17] Samuelsson A., Annerstedt L., Elmstahl S., Samuelsson S.M., Grafström M. (2001). Burden of responsibility experienced by family caregivers of elderly dementia sufferers. Scand. J. Caring Sci..

[18] Hill M., Bramley G. (1992). Analysing Social Policy.

[19] Kontos P.C., Naglie G. (2009). Tacit knowledge of caring and embodied selfhood. Sociol. Health Illn..

[20] Roberts C., Mort M. (2009). Reshaping what counts as care: Older people, work and new technologies. Alter Eur. J. Disabil. Res..

[21] Gadrey L. (1996). L’économie des Services.

[22] Djellal F., Gallouj F. (2006). Innovation in care services for older people. Serv. Ind. J..

[23] Brownsell S., Bradley D., Porteus J., Hawley M. (2003). Assistive Technology and Telecare: Forging Solutions for Independent Living.

[24] Fisk M.F. (2003). Social Alarms to Telecare: Older People’s Services in Transition.

[25] Hanson J., Percival J., Clarkson J., Langdon P., Robinson P. (2006). Differing perspectives on telecare: An attitudinal survey of older people, professional care workers and informal carers. Designing Accessible Technology.

[26] Hanson J., Percival J., Aldred H., Brownsell S., Hawley M. (2007). Attitudes to telecare among older people, professional care workers and informal carers: A preventative strategy or crisis management?. Univers. Access Inf. Soc..

[27] Sánchez-Criado T., López D. (2009). La traducción del cuidado: La teoría del actor-red y el estudio de la interdependencia en la teleasistencia para personas mayores. Estudios de psicología.

[28] López Gómez D. (2005). Aplicación de la teoría del actor-red al análisis espacial de un servicio de teleasistencia domiciliaria. Revista de Antropología iberoamericana.

[29] Mort M., Milligan C., Roberts C., Moser I. (2008). Ageing, Technology and Home Care.

[30] Tirado F., López D., Callén B., Domènech M. (2008). La producción de fiabilidad en entornos altamente tecnificados. Apuntes etnográficos sobre un servicio de teleasistencia domiciliaria. Papeles del CEIC.

[31] López D. (2009). Securizing care: Networks, immediacy and independence in a home telecare service. Athenea Digit..

[32] Mort M., Roberts C., Milligan C. (2009). Ageing, technology and the home: A critical project. Alter Eur. J. Disabil. Res..

[33] Sparrow R., Sparrow L. (2006). In the hands of machines? The future of aged care. Minds Mach..

[34] Novais P., Costa R., Machado J., Neves J. (2009). A memory assistant for the elderly. Intell. Distrib. Comput..

[35] Sixsmith A., Sixsmith J. (2008). Ageing in place in the United Kingdom. Ageing Int..

[36] Rojas Ocaña M.J., Toronjo Gómez A., Rodríguez Ponce C., Rodríguez Rodríguez J.B. (2006). Autonomía y estado de salud percibidos en ancianos institucionalizados. Gerokomos.

[37] Van der Pas S., Koopman-Boyden P., Waldegrave C. (2007). Living arrangements, ageing in place, and wellbeing among older New Zealanders. Enhancing Wellbeing in an Ageing Society: 65–84 Year Old New Zealanders in 2007.

[38] Schaie K.W., Wahl H.W., Mollenkopf H., Oswald F. (2003). Ageing Independently: Living Arrangements and Mobility.

[39] Andrews G.J., Phillips D.R. (2005). Ageing and Place: Perspectives, Policy, Practice.

[40] IMSERSO (2008). Cuadernos: El modelo residencial a debate. Revista sesenta y más.

[41] Cutchin M.P. (2003). The process of mediated aging-in-place: A theoretically and empirically based model. Soc. Sci. Med..

[42] Latour B. (1999). Pandora’s Hope: Essays on the Reality of Science Studies.

[43] Loredo Narciandi J.C. (2009). Sujetos o actantes? El constructivismo de Latour y la psicología constructivista. Aibr. Revista de Antropología Iberoamericana.

[44] Latour B. (1987). Science in Action: How to Follow Scientists and Engineers Through Society.

[45] De Oliveira Texeira M. (2001). A ciência em ação: Seguindo Bruno Latour. História ciências saúde.

[46] Kranzberg M. (1986). Technology and history: “Kranzberg’s Laws”. Technol. Cult..

[47] Vaughn S., Schumm J.S., Sinagub J.M. (1996). Focus Group Interviews in Education and Psychology.

[48] Denzin N.K., Lincoln Y.S. (1998). Collecting and Interpreting Qualitative Materials.

[49] Denzin N. (2001). The reflexive interview and a performative social science. Qual. Res..

[50] Potter J., Hepburn A. (2005). Qualitative interviews in psychology: Problems and possibilities. Qual. Res. Psychol..

[51] Puchta C., Potter J. (2004). Focus Group Practice.

[52] Bardin L. (1996). El Análisis de Contenido.

[53] Fex A., Ek A.C., Söderhamn O. (2009). Self-care among persons using advanced medical technology at home. J. Clin. Nurs..

[54] Coffey A., Atkinson P. (2003). Encontrar el Sentido a Los Datos Cualitativos: Estrategias Complementarias de Investigación.

[55] Geertz C. (1973). The Interpretation of Cultures: Selected Essays.

[56] Callon M., Lascoumes P., Barthe Y. (2001). Acting in an Uncertain World: An Essay on Technical Democracy.

[57] Pierre L. (1995). Qu’est-ce Que Le Virtual?.

[58] Molyneaux V., Butchard S., Simpson J., Murray C. (2011). Reconsidering the term “carer”: A critique of the universal adoption of the term “carer”. Ageing Soc..

[59] Katz S. (2005). Cultural Ageing: Life Course, Lifestyle, and Senior Worlds.

[60] Neugarten B.L. (1999). Los Significados de la Edad.

[61] Reindal S.M. (1999). Independence, dependence, interdependence: Some reflections on the subject and personal autonomy. Disabil. Soc..

[62] Hockey J., James A. (1993). Growing Up and Growing Old: Ageing and Dependency in the Life Course.

[63] Camdessus B. (1995). Crisis Familiares y Ancianidad.

